# Differential Sensitivity to IL-12 Drives Sex-Specific Differences in the CD8+ T Cell Response to Infection

**DOI:** 10.4049/immunohorizons.1800066

**Published:** 2019-04

**Authors:** Kristel Joy Yee Mon, Elizabeth Goldsmith, Neva B. Watson, Jocelyn Wang, Norah L. Smith, Brian D. Rudd

**Affiliations:** * Department of Microbiology and Immunology, Cornell University, Ithaca, NY 14853; † College of Veterinary Medicine and Biomedical Sciences, Colorado State University, Fort Collins, CO 80523

## Abstract

It is well known that males and females respond differently to intracellular pathogens. Females mount a more robust immune response than males, which decreases their susceptibility to infection but comes at the cost of increasing immunopathology. However, the underlying basis for sex-specific differences in the CD8^+^ T cell response to infection remains poorly understood. In this study, we show that female CD8^+^ T cells have an intrinsic propensity to become short-lived effectors, whereas male CD8^+^ T cells give rise to more memory precursor effector cells after murine infection with either a virus (vaccinia virus) or bacteria (*Listeria monocytogenes)*. Interestingly, we found that the propensity of female CD8^+^ T cells to form short-lived effectors is not because they respond to lower amounts of cognate Ag but rather because they have an enhanced capacity to respond to IL-12, which facilitates more effector cell differentiation at each round of cell division. Our findings provide key insights into the sex-based immunological differences that underlie variations in the susceptibility to infection in males and females. *ImmunoHorizons*, 2019, 3: 121–132.

## INTRODUCTION

Numerous studies have demonstrated that sex has an impact on the outcome of infections (1–3). In general, males are more susceptible to a wide variety of pathogens. For example, prevalence rates of leishmaniasis, tuberculosis, and hepatitis A are all significantly higher in males compared with females (4). Also, viral loads are consistently elevated in males infected with hepatitis C virus and HIV (5–7), indicating that females may exhibit an enhanced ability to limit pathogen growth. However, the symptoms of infection are often significantly worse in females compared with males. This can be seen in congenital CMV infection, in which neurologic sequalae are nearly two times higher in females than in males (8). Other studies have indicated that females experience more severe disease after influenza infections, which is believed to relate to increased levels of chemokines and cytokines (9).

Sexual dimorphism in the outcome of infectious diseases likely relates to differences in the immune response during infection. A key component of the host response to intracellular infections involves CD8^+^ T cells. Females tend to have a higher CD8^+^ T cell count than males (10, 11). Female CD8^+^ T cells also exhibit enhanced upregulation of antiviral and proinflammatory genes after in vitro stimulation with PMA–ionomycin (12). These observations provide an initial insight into why females are less vulnerable to intracellular pathogens yet respond with more inflammation. However, sex-specific differences in the CD8^+^ T cell response to infection in vivo remain poorly understood.

Following microbial challenge, naive CD8^+^ T cells are stimulated by mature dendritic cells presenting cognate Ag in the context of MHC classI (13, 14). Although TCR and costimulatory signals initiate proliferation of naive CD8^+^ T cells, inflammatory cytokines (e.g., IL-12) are required for robust effector CD8^+^ T cell differentiation (15). After stimulation with the appropriate signals, naive CD8^+^ T cells undergo massive clonal expansion and differentiate into distinct subsets of effectors, including the short-lived effector cells (SLECs; KLRG1^+^CD127^−^) and memory precursor effector cells (MPECs; KLRG1^−^CD127^+^) (16, 17). SLECs are more terminally differentiated and therefore more apoptotic, express high levels of cytokines and cytolytic molecules, and are largely responsible for eliminating infected cells during infection. MPECs, in contrast, respond less vigorously during infection but retain the ability to transition into the long-lived memory pool and respond to repeat infections with the same pathogen. A key question is whether male and female CD8^+^ T cells undergo different amounts of effector cell differentiation during infection.

Sex-based changes in the CD8^+^ T cell response to infection may be due to environmental and/or cell-intrinsic differences in males and females. In this report, we asked whether cell-intrinsic differences between male and female CD8^+^ T cells influence their fate during infection. To answer this question, we used an experimental strategy whereby equal numbers of male and female CD8^+^ T cells expressing an identical TCR respond to infection in the same host. These studies revealed significant differences in the fates of male and female CD8^+^ T cells driven by their differential sensitivity to IL-12 signaling. Our results provide new insight into the unknown factors underlying sex-related changes in the CD8^+^ T cell response and demonstrate that female cells have an inherent propensity to undergo effector cell differentiation postinfection.

## MATERIALS AND METHODS

### Mice

C57Bl6 B6-Ly5.2/Cr mice were purchased from the National Cancer Institute colony, and B6-Thy1.1/CyJ and IL-12rb2/J KO mice were purchased from The Jackson Laboratory. All mouse strains were crossed with gBT-I TCR transgenic mice (transgenic for TCRαβ specific for the HSV-1 glycoprotein gB_498–505_ peptide SSIEFARL) provided by Dr. J. Nikolich-Zugich (University of Arizona, Tucson, AZ). All mice were used at 8–12 wk of age for experiments and were maintained under pathogen-free conditions at Cornell University’s College of Veterinary Medicine. The experiments in this study were performed in strict accordance with the recommendations in the Guide for the Care and Use of Laboratory Animals of the National Institutes of Health, and the protocols were approved by the Institutional Animal Care and Use Committee at Cornell University.

### Flow cytometry and reagents

mAbs anti-CD8α (53–6.7), anti-CD4 (GK1.5), anti-CD45.1 (A20), anti-CD45.2 (104), anti-CD90.1/Thy1.1 (OX-7), anti-KLRG1 (2F1), anti-CD127 (A7R34), anti-CD62L (MEL-14), anti-CD69 (H1.2F3), anti-Ly6C (HK1.4), anti-CD122 (TM-b1), anti-CD44 (IM7), anti-Tbet (Ebio 4B10), anti–IFN-γ (XMG1.2), anti–granzyme B (GB11), anti–IL-12Rβ2/CD212 (305719), anti–IL-10R/CD210 (1B1.3a), and Fixable Viability Dye were purchased from BioLegend (San Diego, CA), eBioscience (San Diego, CA), Invitrogen (Carlsbad, CA), or R&D Systems (Minneapolis, MN). Fluorochrome-conjugated Kb:gB_498–505_ tetramer was supplied by the National Institutes of Health Tetramer Core Facility. Flow cytofluorimetric data were acquired on a custom FACS LSR II instrument equipped with four lasers, using the DIVA software (BD Biosciences, Mountain View, CA). Analysis was performed using the FlowJo software (Tree Star, Ashland, OR).

### Infections

Mice were i.v. infected with 5 × 10^3^ CFU of either wild-type *Listeria monocytogenes* [strain 10403, obtained from Dr. Nikolich-Zugich (18), designated WT-LM], or a recombinant strain of *L. monocytogenes* expressing the gB peptide, designated LM-gB [obtained from Dr. S. Sing Way (19)]. Unless stated otherwise, mice were inoculated with 5 × 10^3^ CFU of LM-gB. For all experiments, colonies were selected from the plate for growth in liquid culture. Bacteria were grown to log phase, and mice were injected i.v. Recombinant vaccinia virus expressing the gB peptide (VACV-gB) (20) was generously provided by Dr. S.S. Tevethia (Pennsylvania State University, College of Medicine). VACV-gB viral stocks were propagated and quantified in 143B cells (American Type Culture Collection), as previously described (21). Mice were infected with 2 × 10^5^ PFU of VACV-gB (i.p.).

### In vitro proliferation assay

Spleens were harvested from gBT-I mice and pressed through a 40-μm mesh to prepare single-cell suspensions. CD8^+^ T cells were purified using CD803α microbeads (Miltenyi Biotec, Auburn, CA) according to the manufacturer’s protocol. The isolated CD8^+^ T cells were resuspended in PBS and labeled with CFSE, as described previously (21). Cells were resuspended in medium containing exogenous IL-2 (2 ng/ml) and stimulated with either cognate peptide (SSIEFARL) in a 96-well round-bottom plate or plate-bound mouse anti-CD3ε (5 μg/ml) plus anti-CD28 (20 μg/ml) (Invitrogen by Thermo Fisher Scientific) in a 96-well flat-bottom plate. The gating strategy for these flow cytometric data is CD8^+^CD4^−^CD62L^−^CFSE_lo_.

### *In vitro* “*bystander activation*” *assay*

CD8^+^ T cells were resuspended in RPMI 1640 complete medium containing 10% FBS with rIL-12 (eBioscience), IL-18 (2 ng/ml) (Medical and Biological Laboratories), and human IL-2 (2 ng/ml) or human IL-2 alone for 24 h. During the final 4 h, brefeldin A (3 μg/ml) was added. After the culture period, cells were stained for intracellular cytokines and effector molecules using the Intracellular Fixation & Permeabilization Buffer Set (eBioscience). The gating strategy for these flow cytometric data is CD8^+^CD4^−^ Viability Dye^lo^IFN-γ^+^ and CD8^+^CD4^−^Viability Dye^lo^gzmB^+^.

### Dual adoptive cotransfer experiments

Splenocytes were collected from congenically marked C57Bl6, Thy1.1, or gBT-I CD45.2 mice, and CD8^+^ T cells were isolated using negative magnetic selection. Splenocytes were incubated with a mixture of biotinylated Abs (anti-CD4, anti-CD19, anti-CD16/32, anti-Ter119, and anti-MHC class II [BioLegend]) and subsequently incubated with streptavidin-coated microbeads (Miltenyi Biotec). Cells were then passed over an LS magnetic column, according to the manufacturer’s instructions. The 1 × 10^4^ CD8-enriched gBT-I splenocytes (85–95% purity) from each male and female adult donor were combined in a 50:50 equal ratio and adoptively transferred (i.v.) into adult B6-Ly5.2 CD45.1 recipient mice the day before infection. At the indicated days postinfection (dpi), the numbers and phenotype of the donor cells in the blood were determined by flow cytometry. The male and female donor CD8^+^ T cells were distinguished by flow cytometric detection of Thy1.1 and CD45.2. The gating strategy for these in vivo flow cytometric data is CD8^+^CD4^−^CD45.2^+^ CD45.1^−^KLRG1^+^CD127^−^ (SLEC) and CD8^+^CD4^−^CD45.2^+^CD45.1^−^ KLRG1^−^CD127^+^ (MPEC).

### In vitro gB peptide restimulation

Splenocytes from infected mice were restimulated in vitro with 10^−7^ M gB peptide for 4 h at 37°C in the presence of brefeldin A. The male and female donor CD8^+^ T cells were distinguished by their expression of Thy1.1 and CD45.2. The gating strategy for these in vivo flow cytometric data is CD8^+^CD4^−^CD45.2^+^CD45.1^−^.

### Dendritic cell IL-12 immunization

Splenic dendritic cells were prepared as previously described (22). Briefly, C57Bl6 mice were i.p. injected with 5–10 × 10^6^ B16-Flt3 L cells (provided by Stephen Jameson, University of Minnesota). After 10–14 d, when tumors were visible, donor mice were given LPS i.v (2 μg/mouse) to mature dendritic cells. The next day, spleens were harvested, and dendritic cells were purified with CD11c microbeads (Miltenyi Biotec) using positive magnetic selection, according to the manufacturer’s instructions. The isolated CD11c^+^ dendritic cells were incubated with 1 μM SSIEFARL peptide for 4–5 h at 37°C in 10 ml medium containing rGM-CSF (50 ng/ml) and B16 Flt3L-conditioned medium with RPMI 1640 complete medium in a 1:2 ratio, respectively. After the culture period, the cells were washed and injected i.v. into recipient B6-Ly5.2 mice for stimulation of gBT-I donor CD8^+^ T cells, which were adoptively transferred into recipient mice 24 h later. Mice were injected with IL-12 (200 ng) or PBS for 4 d, starting on the day that dendritic cells were transferred. The gating strategy for these in vivo flow cytometric data are CD8^+^CD4^−^CD45.2^+^CD45.1^−^ KLRG1^+^CD127^−^ (SLEC) and CD8^+^CD4^−^CD45.2^+^CD45.1^−^KLRG1^−^ CD127^+^ (MPEC).

### Statistical analysis

Statistical analysis was performed using Prism 7 software (GraphPad). Error bars represent SD unless otherwise noted. Significance was determined by unpaired *t* test or two-way ANOVA, followed by Sidak multiple comparisons test, as indicated in the figure legends. Significance is denoted as follows: ***p < 0.001 and ****p < 0.0001.

## RESULTS

### Female mice generate a larger, more terminally differentiated response to infection

**FIGURE 1.**
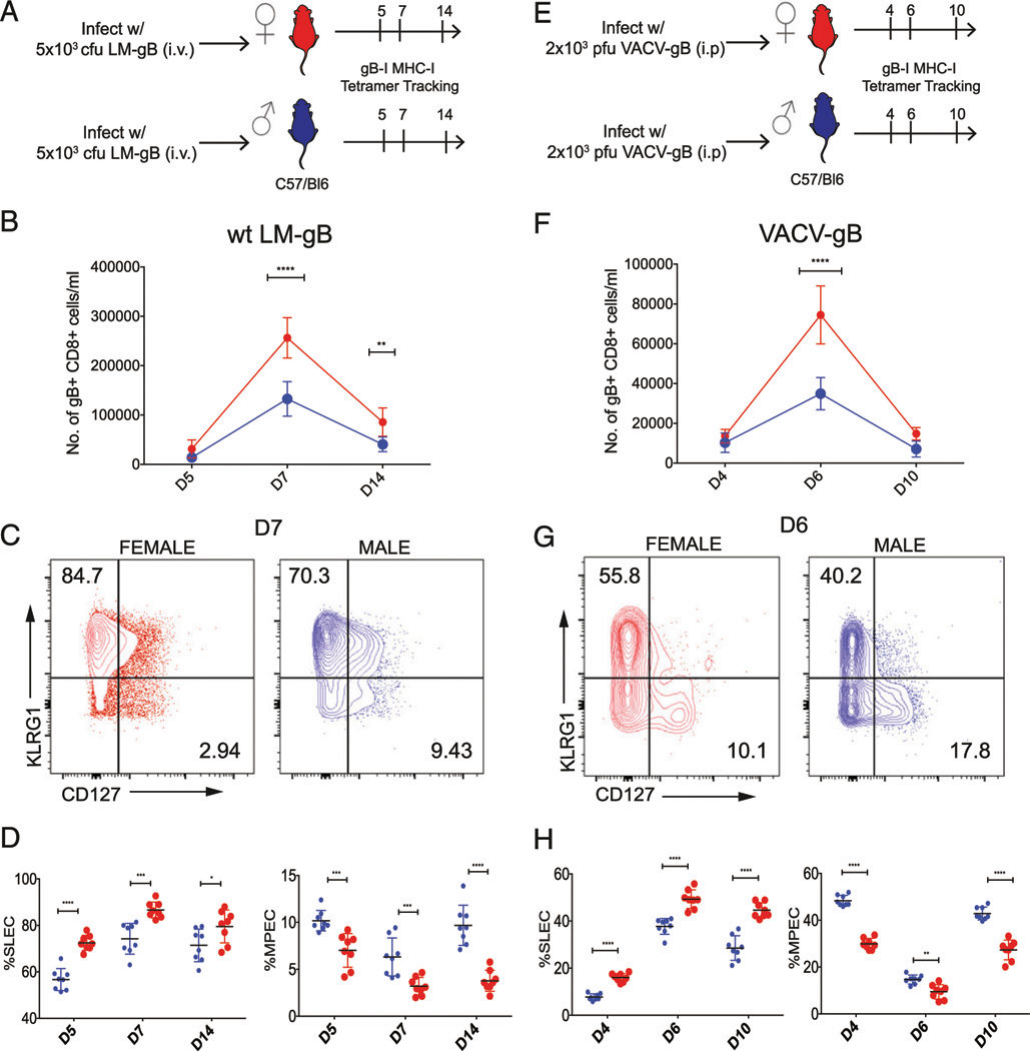
Sex-specific differences in the numbers and phenotype of Ag-specific CD8^+^ T cells postinfection. (**A** and **E**) Schematic of experimental design: female and male C57BL/6 mice were infected with either 5 × 10^3^ CFU of LM-gB or 2 × 10^5^ PFU of VACV-gB and serially bled to monitor gB-specific CD8^+^ T cell responses. (**B** and **F**) Relative numbers of female (red) and male (blue) gB-specific CD8^+^ T cells in the blood at various dpi. (**C** and **G**) Representative contour plots of gB-specific CD8^+^ T cells in male and female mice on day 7 postinfection. Asterisks indicate differences between male and female groups. (**D** and **H**) Percentage of female and male gB-specific CD8^+^ T cells at various dpi that exhibit a SLEC (KLRG1^hi^CD127^lo^) or MPEC (KLRG1^lo^CD127^hi^) phenotype. Significance was determined by two-way ANOVA. Data are representative of two experiments (*n* = 8 mice per group overall). *p < 0.05, **p < 0.01, ***p < 0.001, ****p < 0.0001.

To uncover potential sex-specific differences in the CD8^+^ T cell response to infection, we first systemically infected male and female B6 mice (8–12 wk of age) with a recombinant strain of *L. monocytogenes* that expresses the dominant peptide from the HSV-1 gB glycoprotein (denoted LM-gB). At various stages of infection, we compared the numbers and phenotype of gB-specific CD8^+^ T cells in the blood from both groups of mice using MHC class I tetramers (Fig. 1A). Whereas similar numbers of gB-specific CD8^+^ T cells were detected in both groups of mice during early stages of infection (5 dpi), the overall magnitude of the response at the peak (7 dpi) was significantly larger in female mice (Fig. 1B). We next compared their phenotype at various time points. Interestingly, we found that CD8^+^ T cells in female mice preferentially exhibit a SLEC (KLRG1^+^CD127^−^) phenotype, even at early stages of infection (5 dpi), when the overallcellnumbers are similar (Fig. 1C, 1D). In contrast, CD8^+^ T cells in male mice exhibited more of an MPEC (KLRG1^−^CD127^+^) phenotype throughout the course of infection. These data suggest that females mount a larger CD8^+^ T cell response to infection, which consists of more terminally differentiated effector cells that undergo apoptosis (SLECs).

We next sought to extend our results from a bacterial pathogen to a viral pathogen to assess if this response is pathogen specific. For these experiments, we infected male and female mice systemically with VACV-gB and again tracked Ag-specific CD8^+^ T cells with MHC class I tetramers in the blood (Fig. 1E). Similar to results obtained with LM-gB infections, we observed larger numbers of gB-specific CD8^+^ T cells in female mice (Fig. 1F), which comprised a greater percentage of SLECs (Fig. 1G, 1H). Thus, the female bias toward terminally differentiated effectors (SLECs) does notappear to relate to a specific type of infection but rather to a general sex-specific difference in the CD8^+^ T cell response to acute pathogens.

### Female CD8^+^ T cells have an inherent propensity to adopt a terminally differentiated effector phenotype postinfection

**FIGURE 2.**
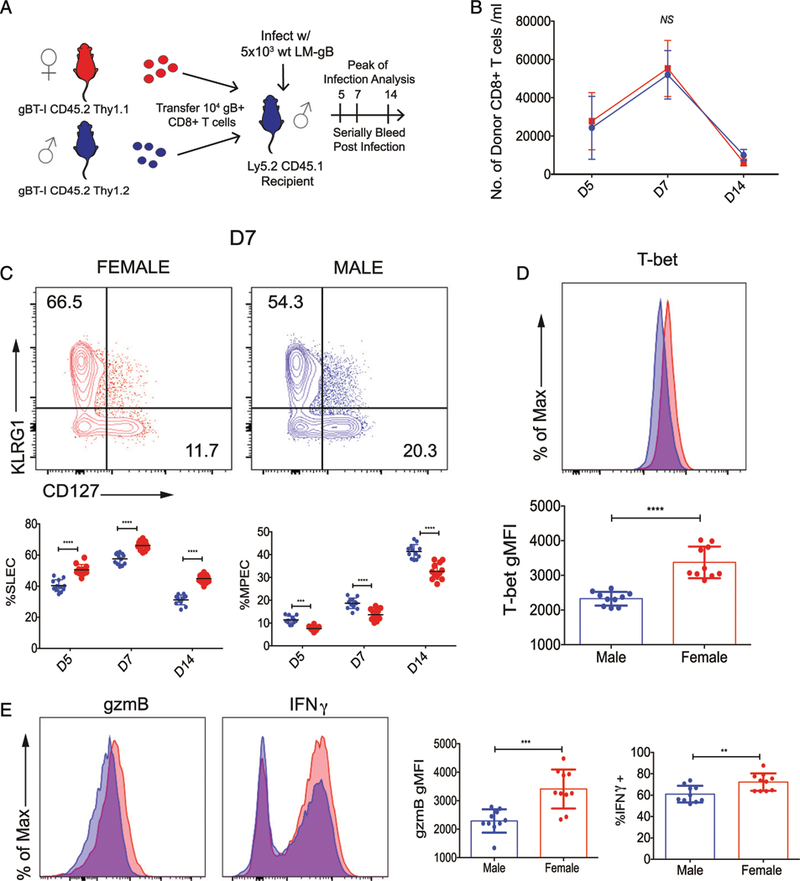
Cell-intrinsic differences between male and female donor CD8^+^ T cells postinfection. (**A**) Schematic of experimental design: 1 × 10^4^ gBT-I CD8^+^ T cells from adult female (Thy1.1, CD45.2) and male (Thy1.2, CD45.2) donors were cotransferred into congenic recipients (Thy1.2, CD45.1). The recipient mice were then infected with 5 × 10^3^ CFU LM-gB and serially bled at days 5, 7, and 14. (**B**) Relative numbers of female (red) and male (blue) donor gBT-I CD8^+^ T cells per ml of blood at days 5, 7, and 14 postinfection. (**C**) Representative contour plots of female and male gB-specific CD8^+^ T cells at the peak of the primary response (day 7) of infection. Percentage of female and male donor gBT-I CD8^+^ T cells at various dpi that exhibit a SLEC (KLRG1^hi^CD127^lo^) or MPEC (KLRG1^lo^CD127^hi^) phenotype. Significance was determined by two-way ANOVA. Data are representative of two experiments (*n* = 12 mice per group overall). (**D**) Representative histogram overlay and geometric mean fluorescence intensity of T-bet expression in male and female donor gB-specific CD8^+^ T cells from spleens of infected mice at 7 dpi. (**E**) Representative histogram overlays and geometric mean fluorescence intensity or percentage of positive gate of cytolytic molecules at 7 dpi. Significance was determined by unpaired *t* test. Data are representative of two experiments (*n* = 10 mice per group overall). **p < 0.01, ***p < 0.001, ****p < 0.0001. NS, *p >* 0.05.

Because we observed similar sex-specific differences in the CD8^+^ T cell response to both pathogens, we suspected that many of the observed phenotypic differences might be due to cell-intrinsic changes in male and female CD8+ T cells. To examine this possibility, we designed an adoptive cotransfer experiment whereby male and female CD8^+^ T cells were able to respond to infection in the same recipient animal, allowing us to control for potential differences in the host environment and focus specifically on cell-intrinsic differences between male and female CD8^+^ T cells (Fig. 2A). The donor CD8^+^ T cells were magnetically isolated from congenically marked TCR transgenic mice that express an H-2Kb–restricted TCR specific for the HSV-1 gB glycoprotein, denoted gBT-I mice (23). Equal numbers (1 × 10^4^) of female (Thy1.1) and male (Thy1.2) gBT-I cells were transferred into male recipient (Ly5.2) mice. The next day, recipient mice were infected with LM-gB, and the donor CD8^+^ T cells were tracked during infection based on their expression of different congenic markers.

Although the numbers of male and female donor CD8^+^ T cells were nearly identical throughout the course of infection (Fig. 2B), the female CD8^+^ T cells still preferentially gave rise to terminally differentiated SLECs, whereas male cells generated more MPECs (Fig. 2C). We also found that T-bet, a transcription factor known to drive effector cell differentiation, was upregulated in female donor CD8^+^ Tcells at the peakofinfection(Fig. 2D). Giventhe phenotypic changes between female and male donor CD8^+^ T cells, we next assessed the expression of cytokines and cytolytic molecules. We observed higher levels of granzyme B and IFN-γ in female donor CD8^+^ T cells at the peak of the response (Fig. 2E), even when comparing phenotype-matched cells directly to each other (Supplemental Fig. 1). Importantly, we did not observe phenotypic differences between male and female CD8^+^ T cells in uninfected gBT-I mice (Supplemental Fig. 2), indicating that the observed phenotypic differences arose postinfection. Collectively, these data demonstrate that female and male CD8^+^ T cells undergo different cell-intrinsic programs of differentiation following microbial challenge.

### Female CD8^+^ T cells preferentially become terminally differentiated across a wide range of antigenic doses

**FIGURE 3.**
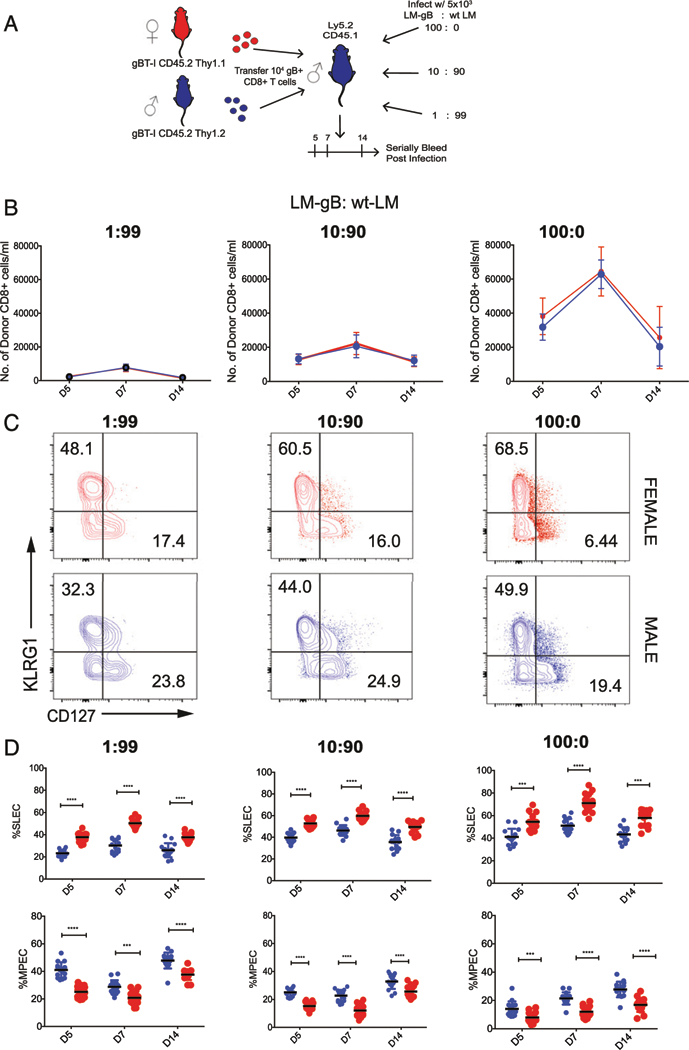
Comparison of male and female CD8^+^ T cells primed with different amounts of cognate Ag. (**A**) Schematic of experimental design: 1 × 10^4^ gBT-I CD8^+^ T cells from female (Thy1.1, CD45.2) and male (Thy1.2, CD45.2) donor mice were cotransferred into Ly5.2 recipient mice (Thy1.2, CD45.1). These recipients were then infected with 5 × 10^3^ CFU LM-gB mixed with 5 × 10^3^ CFU *L. monocytogenes* at the indicated ratios and serially bled at days 5, 7, and 14. (**B**) Relative numbers of adult female (red) and male (blue) donor gBT-I CD8^+^ T cells per ml of blood in mice infected with different amounts of LM-gB at various dpi. (**C**) Representative contour plots of female and male gB-specific CD8^+^ T cells at the peak of the primary response (day 7) of infection. (**D**) Percentage of female and male donor gBT-I CD8^+^ T cells in different treatment groups at various dpi that exhibit a SLEC (KLRG1^hi^CD127^lo^) or MPEC (KLRG1^lo^CD127^hi^) phenotype. Significance was determined by two-way ANOVA. Data are representative of two experiments (*n* = 15 mice per group). ***p < 0.001, ****p < 0.0001.

We next sought to determine why female CD8^+^ T cells have a greater propensity than male cells to become SLECs postinfection. One possible explanation is that they are more sensitive to cognate Ag. To test this, we asked whether we would observe more pronounced sex-specific differences when female and male cells are stimulated with low amounts of cognate Ag. For these studies, we repeated our cotransfer experiment with male and female donor CD8^+^ T cells from gBT-I mice. However, this time, we infected recipient mice with an equivalent number of bacteria containing 100% cognate Ag (1 × 10^4^ LM-gB), 10% cognate Ag (1 × 10^3^ LM-gB plus 9 × 10^3^ *L. monocytogenes*), or 1% cognate Ag (1 × 10^2^ LM-gB plus 9.9 × 10^3^ *L. monocytogenes*) (Fig. 3A). This strategy allows the amount of cognate Ag to be decreased with constant bacteria load and degree of inflammation. On days 5, 7, and 14 postinfection, we bled the mice and compared the numbers and phenotype of the donor cells. Consistent with our previous adoptive transfer experiment, female CD8^+^ T cells were numerically similar to male CD8^+^ T cells throughout the course of infection (Fig. 3B) but exhibited a more terminally differentiated SLEC phenotype at each time point (Fig. 3C, 3D). Importantly, these phenotypic differences were maintained independent of antigenic load, indicating that the propensity of female CD8^+^ T cells to terminally differentiate is not linked to differential Ag sensitivity.

### Female CD8^+^ T cells are highly sensitive to IL-12 stimulation

Another possible explanation for the female bias toward terminally differentiated effector cells is that female CD8^+^ T cells are more sensitive to inflammatory cues. If female CD8^+^ T cells have a lower threshold for activation by inflammatory cytokines, we would expect to observe enhanced functionality after in vitro stimulation with IL-12. To investigate this possibility, we cultured CD8^+^ T cells from uninfected female and male gBT-I mice with IL-12 and IL-18, cytokines that have previously been shown to stimulate the secretion of IFN-γ and gzmB from memory phenotype CD8^+^ T cells (24). After a 24 h pulse, we performed intracellular cytokine staining for IFN-γ and gzmB. We found that, when cultured with IL-2 or IL-18 stimulus alone, CD8^+^ T cells from female and male mice expressed similarly low levels of effector molecules. However, with the addition of IL-12 stimulus, we observed significantly higher levels of IFN-γ and gzmB expression in female CD8^+^ T cells (Supplemental Fig. 3).

**FIGURE 4.**
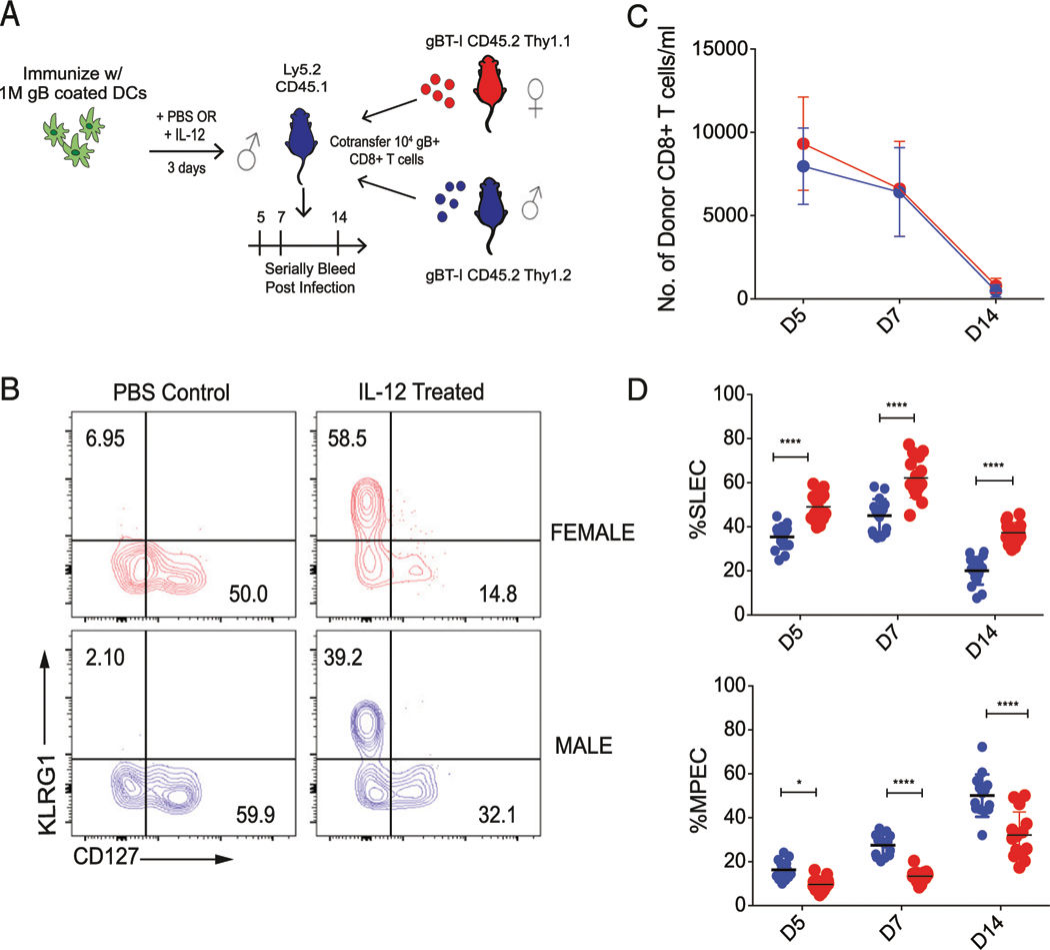
Analysis of male and female CD8^+^ T cells primed in vivo with an equivalent amount of IL-12. (**A**) Schematic of experimental design: 1 × 10^4^ gBT-I CD8^+^ T cells from congenic adult female (Thy1.1, CD45.2) and male (Thy1.2, CD45.2) donors were cotransferred into Ly5.2 recipients (Thy1.2, CD45.1). These recipients were then immunized with 1 × 10^6^ matured dendritic cells coated with gB peptide (i.v.). Recipients were injected with IL-12 or PBS (control) i.p. for three consecutive days and serially bled at days 5, 7, and 14. (**B**) Representative contour plots of female and male gB-specific CD8^+^ T cells at the peak of the primary response (day 7) of IL-12 immunization. (**C**) Relative numbers of adult female (red) and male (blue) donor gBT-I CD8^+^ T cells per milliliter of blood at days 5, 7, and 14 postimmunization. (**D**) Percentage of female and male donor gBT-I CD8^+^ T cells at various days postimmunization in the IL-12–treated group that exhibit a SLEC (KLRG1^hi^CD127^lo^) or MPEC (KLRG1^lo^CD127^hi^) phenotype. Significance was determined by two-way ANOVA. Data are representative of two experiments (*n* = 16 mice per group). *p < 0.05, ****p < 0.0001.

Our in vitro findings prompted us to examine the effects of proinflammatory cytokines on male and female CD8^+^ T cells stimulated in vivo. Previous studies have shown that differentiation to the SLEC phenotype is driven by inflammatory cytokines, such as IL-12 (25). For our studies, we used a dendritic cell vaccination approach to control the amount of IL-12, although levels of cognate Ag remained constant, as previously described (22, 26) (Fig. 4A). We adoptively transferred male and female gBT-I cells into recipient mice and stimulated them with splenic dendritic cells loaded with cognate peptide (SSIEFARL) in the presence of IL-12 (4 daily injections with 200 ng) or PBS (control injections) (Fig. 4A). The numbers and phenotype of the donor CD8^+^ T cells were examined on days 5, 7, and 14 after dendritic cell transfer. At every time point, the female donor CD8^+^ T cells exhibited a more terminally differentiated phenotype compared with their male counterparts (Fig. 4C, 4D). These phenotypic differences do not appear to be because of changes in proliferation as there was no significant difference in cell numbers between the two groups throughout the time course (Fig. 4B). These data indicate that changes in IL-12 stimulation alone are sufficient to recapitulate many of the sex-specific differences in the CD8^+^ T cell response to infection.

**FIGURE 5.**
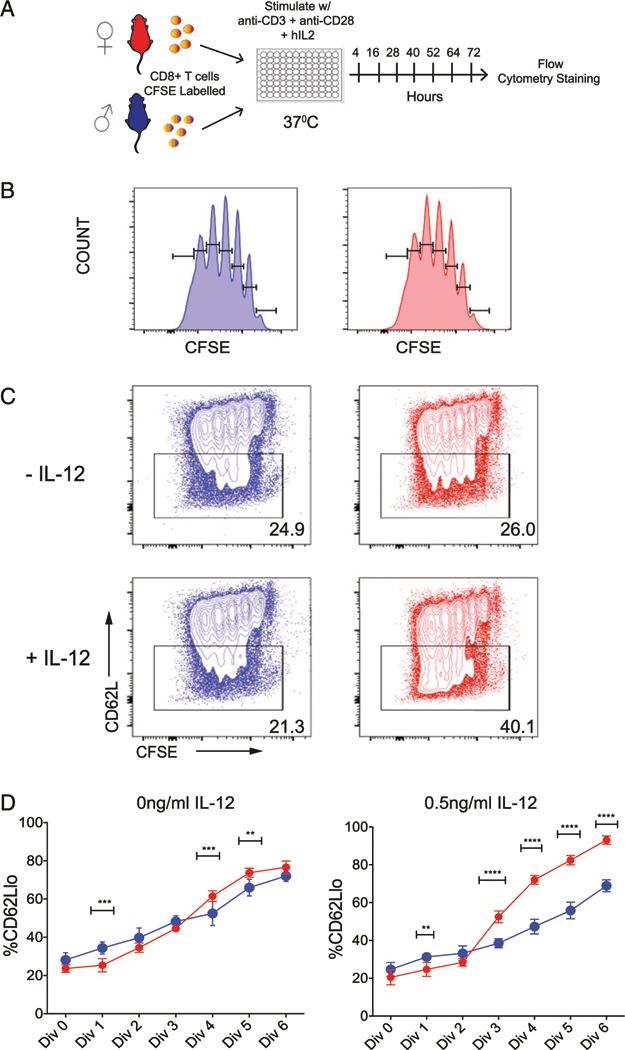
Comparison of male and female CD8^+^ T cells stimulated in vitro. (**A**) Schematic of experimental design: gBT-I CD8^+^ T cells from male and female mice were coated with CFSE and stimulated in vitro with plate-bound anti-CD3 and soluble CD28 in the absence or presence of IL-12. (**B**) Representative CFSE histograms of female and male CD8^+^ T cells at 72 h after TCR stimulation in vitro. (**C**) Representative contour plots of female and male gB-specific CD8^+^ T cells that are labeled in CFSE proliferation dye and express CD62L at 72 h after TCR stimulation in the absence or presence of IL-12. (**D**) Percentage of female and male CD8^+^ T cells that are CD62L^lo^ in the absence or presence of IL-12 at 72 h after TCR stimulation. Significance was determined by two-way ANOVA. Data are representative of two experiments (*n* = 8 mice per group). Asterisks indicate differences between male and female groups. **p < 0.01, ***p < 0.001, ****p < 0.0001.

If male and female CD8^+^ T cells exhibit similar proliferative programs following stimulation with IL-12, why do female CD8^+^ T cells display a more terminally differentiated SLEC phenotype? One possibility is that female cells are proliferating and dying faster than male cells. Alternatively, female cells may be undergoing more differentiation per division than their male counterparts. To distinguish between these possibilities, we isolated CD8^+^ T cells from the spleens of female and male gBT-I mice, labeled the cells with CFSE, and activated them via the TCR (anti-CD3/anti-CD28) in the presence or absence of IL-12 (Fig. 5A). In this way, we could directly study the relationship between cell division and differentiation. Three days after stimulation, we found that female and male CD8^+^ T cells had undergone similar amounts of proliferation as determined by the amount of CFSE dilution (Fig. 5B). We also assessed the rate of differentiation by measuring the proportion of cells with a CD62L^lo^ phenotype. Importantly, this marker has previously been shown to be downregulated at each division at a constant rate (27–29). We found that female and male CD8^+^ T cells downregulated CD62L at a comparable rate after TCR stimulation, but with the addition of IL-12, female CD8^+^ T cells downregulated significantly more CD62L per division (Fig. 5B, 5C). For example, nearly 70% of female CD8^+^ T cells lost CD62L after four divisions compared with only 40% of male CD8^+^ T cells. These data demonstrate that female CD8^+^ T cells undergo more division-linked differentiation than male CD8^+^ T cells upon stimulation with IL-12.

### Female CD8^+^ T cells are more dependent on IL-12 during infection

**FIGURE 6.**
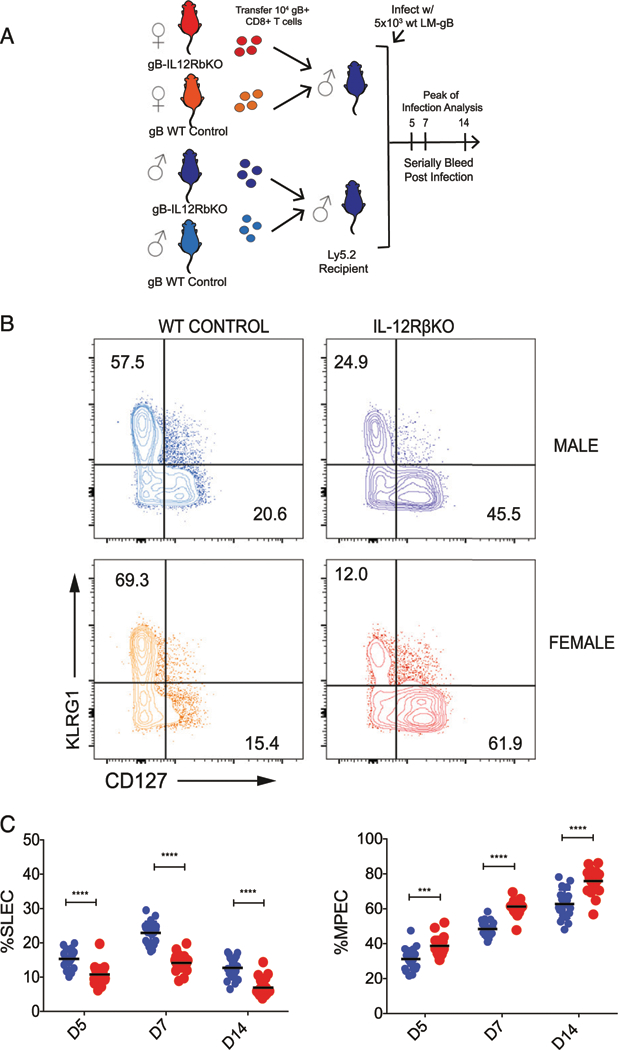
Sex-specific differences in the phenotype of IL-12–deficient CD8^+^ T cells postinfection. (**A**) Schematic of experimental design: gBT-I CD8^+^ T cells from congenic female or male control mice (Thy1.1, CD45.2) and male or female IL-12Rβ KO (Thy1.2, CD45.2) donors were cotransferred into Ly5.2 recipients (Thy1.2, CD45.1). Recipient mice were then infected with 5 × 10^3^ CFU LM-gB and serially bled at days 5, 7, and 14. (**B**) Representative contour plots of female and male donor gB-specific CD8^+^ T cells at the peak of the primary response (day 7) of infection. (**C**) Percentage of female and male donor gBT-I IL-12Rβ KO CD8^+^ T cells at various days postimmunization that exhibit a SLEC (KLRG1^hi^CD127^lo^) or MPEC (KLRG1^lo^CD127^hi^) phenotype. Significance was determined by two-way ANOVA. Data are representative of two experiments (*n* = 20 mice per group). ***p < 0.001, ****p < 0.0001.

If differential sensitivity to IL-12 underlies sex-specific differences in the CD8^+^ T cell response to infection, then male and female CD8^+^ T cells should exhibit a similar phenotype in the absence of IL-12. To examine this possibility, we crossed our gBT-I mice with mice that were deficient in IL-12RB1 to generate a source of donor CD8^+^ T cells incapable of responding to IL-12 (denoted IL-12R KO). We then adoptively cotransferred male or female IL-12R KO gBT-I cells into male congenic recipient mice, along with an equivalent number of male or female wild-type Thy1.1 gBT-I cells to serve as a reference population in each animal (Fig. 6A). The next day, we infected the recipient mice with LM-gB and compared the numbers and phenotype of each group of donor CD8^+^ T cells at various times postinfection. Both male and female CD8^+^ T cells lacking IL-12 signaling gave rise to significantly fewer SLECs than wild-type controls at 7 dpi (Fig. 6B). When we directly compared male and female IL-12R KO cells, we observed similar numbers of donor CD8^+^ T cells throughout the course of infection (data not shown). However, male IL-12R KO cells differentiated into more SLECs and fewer MPECs than their female counterparts (Fig. 6B, 6C, Supplemental Fig. 4A, 4B), which may be linked to a more pronounced reduction in the level of IL-10R expression (Supplemental Fig. 4C, 4D) (30). Collectively, these results suggest that IL-12 signaling is required for female CD8^+^ T cells to preferentially differentiate into SLECs during infection.

## DISCUSSION

In this study, we performed a series of adoptive transfer experiments to study sex-specific differences in effector CD8^+^ T cell differentiation. We showed that female donor CD8^+^ T cells preferentially become SLECs even when primed in the same host as male CD8^+^ T cells. The female bias toward the SLEC lineage was present in recipient mice across a wide range of doses, which prompted us to compare the ability of male and female CD8^+^ T cells to respond to cytokines. We demonstrated, using dendritic cell vaccination studies, that female CD8^+^ T cells become more terminally differentiated in the presence of IL-12. Interestingly, the increase in effector cell differentiation by female cells was not due to an increase in their expansion after stimulation. Instead, our in vitro analysis demonstrated that female CD8^+^ T cells undergo more differentiation per division in the presence of IL-12. Also, female CD8^+^ T cells that lack IL-12 signaling no longer retain their inherent propensity to become terminally differentiated during infection. Thus, female CD8^+^ T cells more quickly become terminally differentiated postinfection because they are intrinsically more responsive to IL-12.

Given that female mice elicited a significantly larger number of tetramer^+^CD8^+^ T cells at the peak of infection (Fig. 1), we were surprised to discover that female and male donor CD8^+^ T cells undergo a similar amount of expansion in our adoptive transfer experiments (Fig. 2). One potential explanation for this difference is that female mice possess an enhanced ability to prime CD8^+^ T cells. Indeed, previous studies have indicated that certain pattern-recognition receptors (e.g., TLR7) are more abundantly expressed in female dendritic cells (30, 31). APC in females also upregulate more MHC molecules than their male counterparts after stimulation (32), and estradiol has been shown to increase IL-12 production in splenic dendritic cells (33, 34). Thus, it is possible that a more robust innate immune response in females contributes to a larger and more differentiated endogenous CD8^+^ T cell response postinfection. Unfortunately, we were unable to test this possibility because of the rejection of male donor CD8^+^ T cells in female recipient mice.

Whereas males are generally more vulnerable to infection, numerous studies have reported a female bias toward inflammatory and autoimmune diseases. For instance, systemic lupus erythematosus, scleroderma, and rheumatoid arthritis are all more commonly found in females than males (35). Although the underlying basis for why women are more prone to autoimmunity is poorly understood, it is intriguing to speculate that sex-related differences in the bystander activation of CD8^+^ T cells may be a contributing factor. In this study, we found that female CD8^+^ T cells secrete more IFN-γ and gzmB after stimulation with innate cytokines alone. Importantly, the differential production of cytokines/cytolytic molecules by female and male CD8^+^ T cells are expected to be even more pronounced during chronic conditions because CD8^+^ T cells are repeatedly stimulated with proinflammatory cytokines, which may lead to additional positive feedback loops.

Although the underlying basis for enhanced SLEC differentiation in female CD8^+^ T cells is unknown, several possibilities are worth mentioning. First, a number of proinflammatory genes (IFN-γ, gzmB) are known to be more responsive to estrogen, which is found at higher concentrations in female mice. There is also an estrogen response element in the promoter region of IL-12Rβ that could contribute to enhanced IL-12 signaling in female CD8^+^ T cells (12). Interestingly, our experiment with IL-12R KO donor cells suggests that female CD8^+^ T cells are not only more responsive to IL-12 but also more dependent on IL-12 signaling during infection. Indeed, in the absence of IL-12 signaling, female CD8^+^ T cells express more IL-10R and form significantly fewer SLECs than their male counterparts. Second, male and female CD8^+^ T cells contain different sex chromosomes, and a number of X-linked genes are known to promote effector cell differentiation. For example, CXCR3 is an X-linked gene that has previously been shown to position cells in regions of the lymph node that receive greater amounts of antigenic stimulation, resulting in an outgrowth of SLECs at the expense of memory CD8^+^ T cell development (36). Clearly, further studies are needed to test these possibilities and better understand how sex hormones and chromosomes alter the CD8^+^ T cell response to infection.

In summary, our findings shed new light on how effector cell differentiation differs in female and male CD8^+^ T cells. Our findings demonstrate that female CD8^+^ T cells are skewed toward effector cell differentiation in a cell-intrinsic manner and may need to be taken into consideration in the development of therapeutics to boost or restore T cell immunity in males and females.

## Supplementary Material

1

## Abbreviations used in this article:

dpi: day postinfection
LM-gB: recombinant strain of *L. monocytogenes* expressing the gB peptide
MPEC: memory precursor effector cell
SLEC: short-lived effector cell
VACV-gB: recombinant vaccinia virus expressing the gB peptide
