# Supplementary material for: Differential Sensitivity to IL-12 Drives Sex-Specific Differences in the CD8+ T Cell Response to Infection
Source: Immunohorizons. Author manuscript; Available in PMC 2019 Jul 17. (PMC6636834; doi:10.4049/immunohorizons.1800066)
Supplement: 1 [file NIHMS1022539-supplement-1.pdf]

SUPPLEMENTARY FIGURE 1

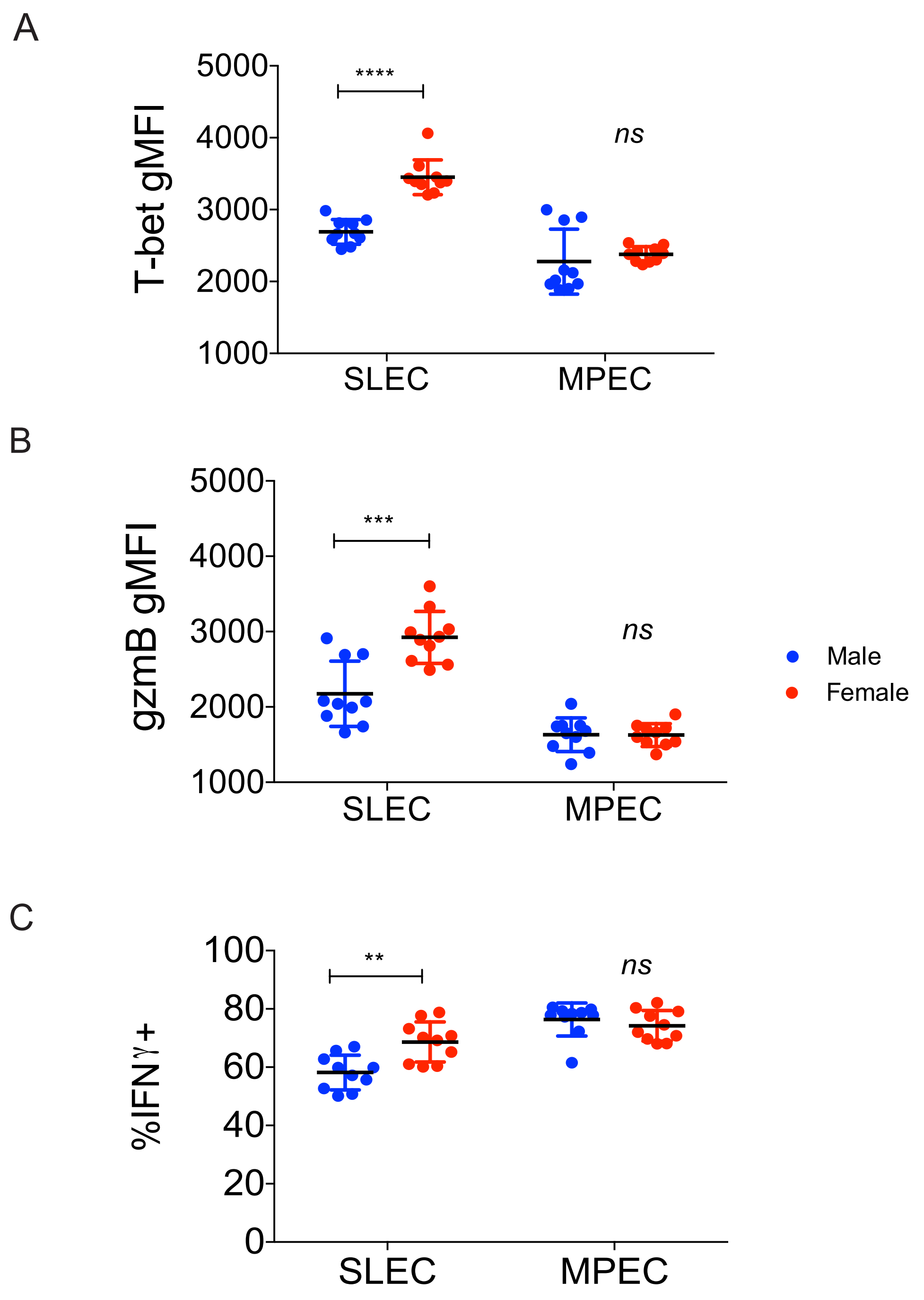

Figure S1: ***Female short-lived effector CD8+ T cells express higher levels of T-bet and secrete more effector molecules upon peptide restimulation.***

(A) Mean geometric fluorescence intensity of T-bet in female and male gBT-I cells at 7 days after infection with LM-gB.

(B-C) Mean geometric fluorescence intensity of pro-inflammatory cytokine expression in female and male gBT-I cells that were restimulated in vitro with gB peptide for 4 hours at 37°C. Significance was determined by unpaired t-test. Data are representative of 2 experiments (n = 10 mice/group). ****p, 0.0001.

SUPPLEMENTARY FIGURE 2

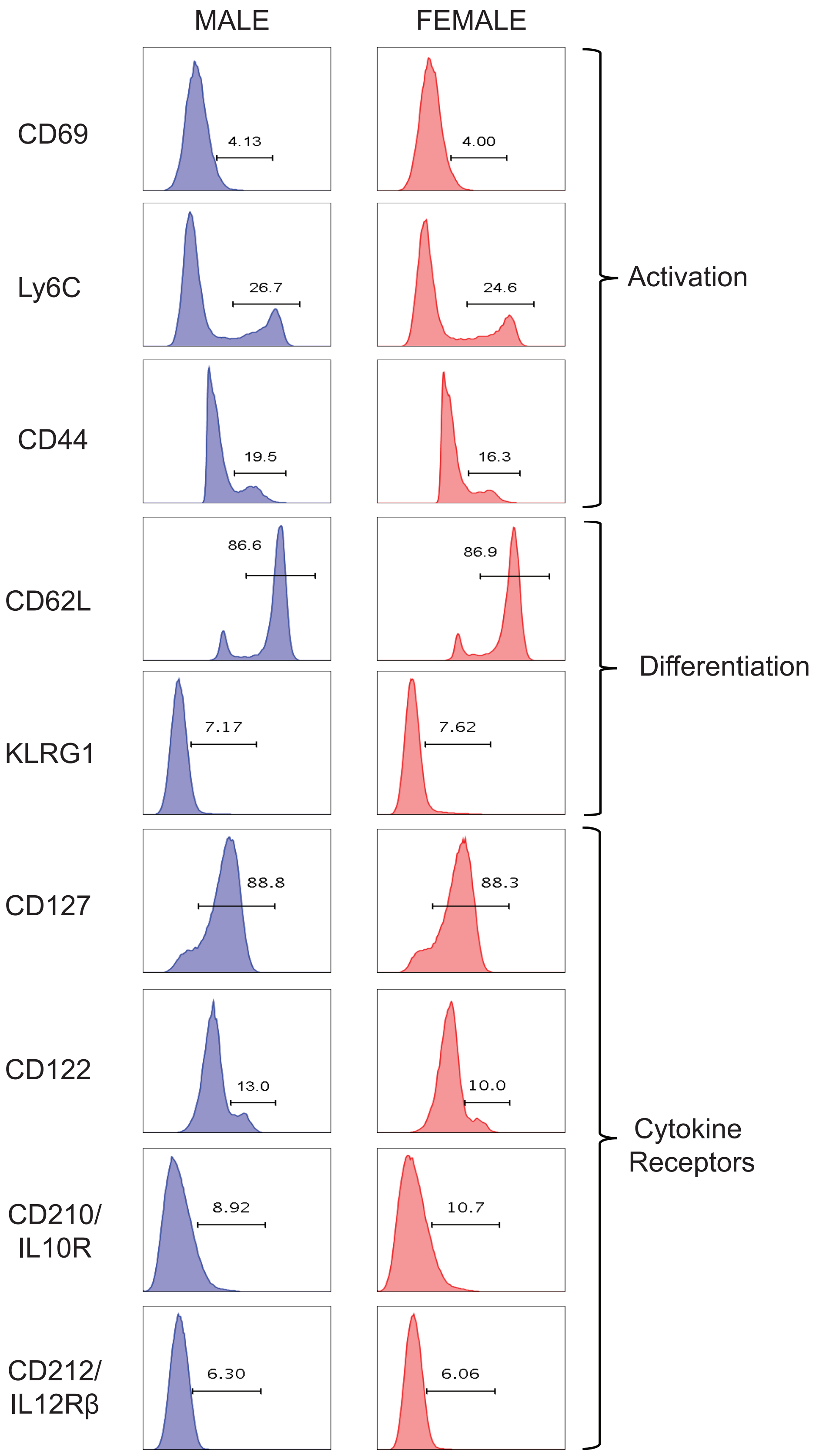

Figure S2*: CD8+ T cells in uninfected male and female gBT-I mice exhibit a similar phenotype.* Representative histograms of markers associated with activation and differentiation expressed in CD8+ T cells obtained in the blood of male or female gBT-I mice. Data are representative of 2 experiments (n = 10 mice/group).

SUPPLEMENTARY FIGURE 3

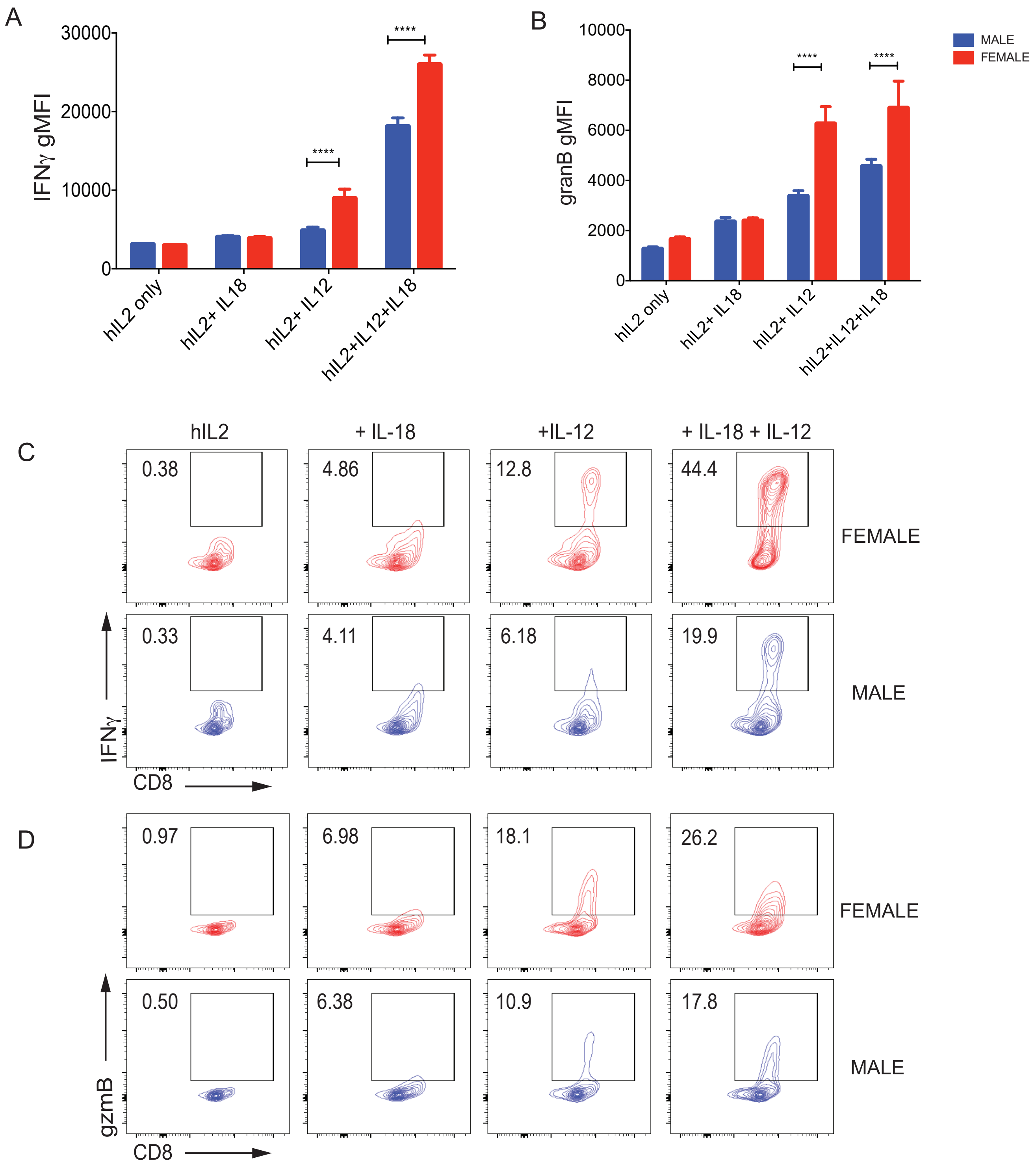

Figure S3***: Female CD8+ T cells secrete more effector molecules upon proinflammatory cytokine stimulation.***

(A-B) Mean geometric fluorescence intensity of female and male gBT-I cells that were stimulated in vitro by IL-2 (control) or with various combinations of IL-2, IL-12 and IL-18 for 18 hours.

(C-D) Representative flow cytometric contour plots of CD8+ T cells expressing IFNγ and gzmB proinflammatory cytokine. Significance was determined by 2 Way ANOVA. Data are representative of 2 experiments (n = 8 mice/group). ****p, 0.0001.

SUPPLEMENTARY FIGURE 4

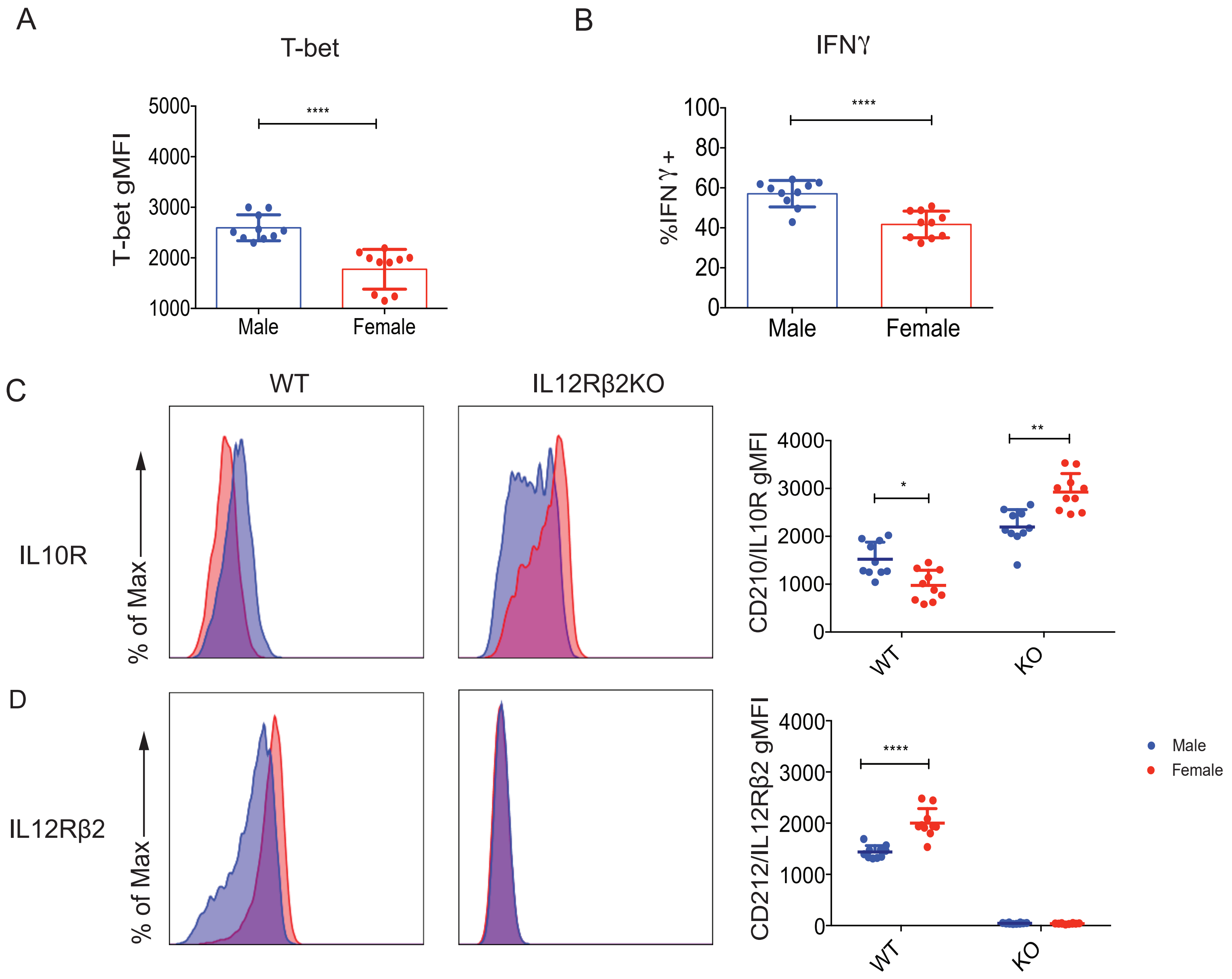

Figure S4: ***Female CD8+ T cells express less IL10R and more IL12R compared to male counterparts during infection.***

(A) Geometric mean fluorescence intensity of T-bet expression in male and female donor gB-specific IL12Rβ-KO CD8+ T cells from spleens of LM-gB infected mice at 7 days post infection.

(B) Percent positive IFNγ gate of male and female donor gB-specific IL12Rβ-KO CD8+ T cells from spleens of LM-gB infected mice at 7 days post infection after gB restimulation. Significance was determined by unpaired t test. Data are representative of 2 experiments (n =10 mice/ experiment per group overall) ****p, 0.0001.

(C-D) Representative histogram overlay and mean geometric fluorescence intensity of IL10R and IL12Rβ2 expression in female and male WT and IL12Rβ-KO gBT-I cells at Day 7 of LM-gB infection. Significance was determined by unpaired t test. Data are representative of 2 experiments (n = 10 mice/group). ****p, 0.0001.
